# Massachusetts Veterinary Association

**Published:** 1895-01

**Authors:** John M. Parker

**Affiliations:** Secretary


### SOCIETY PROCEEDINGS.

### MASSACHUSETTS VETERINARY ASSOCIATION.

THE monthly meeting of the Massachusetts Veterinary Association was held on September 12, 1894, at 19 Boylston Place. The members present were Drs. Becket, Burr, Fowle, Howard, Labaw, McKenna, Osgood, Parker, Winchester, and Winslow. Honorary member, Dr. Stickney. The chair was taken by Dr. Burr at 7.45 P.M.

The minutes of the last meeting having been read, Dr. Winslow moved that they be accepted as read; seconded by Dr. Howard. Carried.

The Secretary then read the report of the Committee on Tuberculosis. Dr. Winchester moved that the report be accepted; seconded by Dr. Osgood. Carried.

Dr. Winchester then said he had looked over the report, and thought it covered the ground; the points were well taken. He thought it should be published in journals, and he saw no reason why it should not be adopted and published.

Dr. Howard thought it pretty complete, and should be published in the agricultural papers.

Dr. Burr then suggested that the report should be considered in parts.

Dr. Osgood was of the opinion that the matter of disinfection should be treated a little more fully.

After some further discussion Dr. Osgood moved that the report be printed and copies mailed to the members of the Association, and the matter brought up at the next meeting; seconded by Dr. Stickney. Carried.

Applications for membership were then received from Dr. James T. Fisher, 98 Moreland street, Boston; Dr. Henry Lewis, Chelsea; and Dr. H. B. Hamilton, Albany street, Boston. The names of Dr. Lemuel Pope, Arlington Heights, and Dr. J. H. Walker, Taunton, were then brought up, and as no applications from them had been received, the Secretary was directed to forward application blanks to them.

The Committee on Credentials then reported favorably on the names of Drs. Lewis and Fisher.

*New Business.* Dr. Burr then called attention to the fact that members of committees are sometimes compelled to travel long distances to attend committee meetings, and he suggested that Dr. Paige be remunerated for expenses incurred in attending committee meetings in Boston. After considerable discussion the general opinion seemed to be that it was not advisable to establish a precedent by paying the expenses of those attending committee meetings.

Dr. Osgood moved that this meeting be considered the regular September meeting of the Association, and that the regular meeting of the Association be held on the fourth Wednesday of each month; seconded by Dr. Labaw and carried.

Dr. Osgood gave written notice that at the next meeting he would propose an amendment to the Constitution to provide for special meetings.

Dr. Parker then called attention to the meeting of the United States Veterinary Medical Association in Philadelphia, and asked that the Association should appoint delegates to attend the Philadelphia meeting.

Dr. Osgood moved that all those attending the meeting should be appointed as delegates; seconded by Dr. Parker. Carried.

The matter of spaying cows was then brought up for discussion by Dr. Osgood reading a letter relating to the spaying of cows. He had been doing a good deal of it lately, and so far the results have been satisfactory, although the time has not been sufficiently long to give the matter a thorough test. He has spayed about forty cows. In his experience cows are apt to gain in flesh unless carefully watched, and where they gain in flesh and get fat the feed should be dropped.

After some further discussion the meeting adjourned.

JOHN M. PARKER,
Secretary.

THE regular monthly meeting of the Massachusetts Veterinary Association was held at No. 19 Boylston Place, on Wednesday, October 24, 1894, at 7.30 P.M. The members present were Drs. Bunker, Emerson, Howard, Labaw, Osgood, Parker, Rogers, Winchester, and Winslow. Dr. Winchester occupied the chair.

The minutes of the last meeting having been read and adopted, the Chair called on Dr. Howard, as one of the delegates to the United States Veterinary Medical Association meeting in Philadelphia, to make his report.

Dr. Howard made an interesting report of the meeting. He referred to the report of the Committee on Education, to Dr. Harger's paper on "Neurotomy" or "Neurectomy," and to the papers on tuberculosis, as being especially interesting. In reply to an inquiry, he stated that the Comitia Minora recommended the expulsion of Drs. Lee and Becket in consequence of having sent out illuminated calendars. The Association finally decided to call the attention of Drs. Lee and Becket to the matter, so that they might have an opportunity to defend themselves.

The meeting closed on the last day with a most enjoyable visit to the Veterinary Department of the University of Pennsylvania, to the hospital of Dr. W. Horace Hoskins, and to the Zoological Gardens, finishing with dinner at the Colonnade Hotel.

Dr. Winchester, Dr. Osgood, and Dr. Parker, all reported having enjoyed the meeting very greatly.

Dr. Parker then reported for the Committee on Tuberculosis; after considerable discussion the report of the committee was accepted as one of progress, and certain changes were advocated in the report.

The names of Dr. James T. Fisher and Dr. Henry Lewis were then voted on, and unanimously elected to membership, and the names of Dr. H. B. Hamilton and Dr. Leonard Pope were reported on favorably and laid on the table for action at the next meeting.

*New Business.*—The Secretary then read a letter from Dr. Lee resigning from membership in the Association. Dr. Winslow moved that the resignation be accepted; Dr. Bunker moved an amendment "that Dr. Lee's resignation be laid on the table, and the Secretary instructed to write to Dr. Lee asking him why, in the event of his resignation not being accepted, he requests to be expelled." Seconded and adopted.

Dr. Osgood then brought up the matter of special meetings, and after some discussion Dr. Bunker made the following motion: that "special meetings can only be called by the President or upon written application of three or more members, or by vote of the Association." Seconded by Dr. Osgood and carried unanimously.

Dr. Osgood then informed the Association that in consequence of the generosity of Messrs. Hemenway, of Boston, and Morgan, of New York, Harvard Veterinary College is now enabled to do away with the subscription plan.

After some further discussion Dr. Bunker offered a resolution approving of their action, and by vote of the Association the consideration of the resolution was laid over till the next meeting.

There being no further business the meeting adjourned till November.

JOHN M. PARKER,
Secretary.

